# The Different Roles of Glucocorticoids in the Hippocampus and Hypothalamus in Chronic Stress-Induced HPA Axis Hyperactivity

**DOI:** 10.1371/journal.pone.0097689

**Published:** 2014-05-15

**Authors:** Li-Juan Zhu, Meng-Ying Liu, Huan Li, Xiao Liu, Chen Chen, Zhou Han, Hai-Yin Wu, Xing Jing, Hai-Hui Zhou, Hoonkyo Suh, Dong-Ya Zhu, Qi-Gang Zhou

**Affiliations:** 1 Department of Pharmacology, Pharmacy College, Nanjing Medical University, Nanjing, China; 2 College of Pharmacy, Nanjing University of Chinese Medicine, Nanjing, China; 3 Lerner Research Institute, Cleveland Clinic, Cleveland, Ohio, United States of America; 4 Institute of Neuroscience, Coochow University, Su zhou, China; 5 The affiliated suzhou hospital of nanjing medical university, Su zhou, China; 6 School of Applied Science, Temasek Polytechnic, Singapore, Singapore; Radboud University, Netherlands

## Abstract

Hypothalamus-pituitary-adrenal (HPA) hyperactivity is observed in many patients suffering from depression and the mechanism underling the dysfunction of HPA axis is not well understood. Chronic stress has a causal relationship with the hyperactivity of HPA axis. Stress induces the over-synthesis of glucocorticoids, which will arrive at all the body containing the brain. It is still complicated whether glucocorticoids account for chronic stress-induced HPA axis hyperactivity and in which part of the brain the glucocorticoids account for chronic stress-induced HPA axis hyperactivity. Here, we demonstrated that glucocorticoids were indispensable and sufficient for chronic stress-induced hyperactivity of HPA axis. Although acute glucocorticoids elevation in the hippocampus and hypothalamus exerted a negative regulation of HPA axis, we found that chronic glucocorticoids elevation in the hippocampus but not in the hypothalamus accounted for chronic stress-induced hyperactivity of HPA axis. Chronic glucocorticoids exposure in the hypothalamus still exerted a negative regulation of HPA axis activity. More importantly, we found mineralocorticoid receptor (MR) - neuronal nitric oxide synthesis enzyme (nNOS) - nitric oxide (NO) pathway mediated the different roles of glucocorticoids in the hippocampus and hypothalamus in regulating HPA axis activity. This study suggests that the glucocorticoids in the hippocampus play an important role in the development of HPA axis hyperactivity and the glucocorticoids in the hypothalamus can't induce hyperactivity of HPA axis, revealing new insights into understanding the mechanism of depression.

## Introduction

Major depressive disorder (MDD) is a severe, life threatening, and highly prevalent mood disease. It ranks the most common of all psychiatric disorders [1]. Despite the fact that several hypotheses have been postulated, the exact mechanisms underlying MDD remain not clear. Chronic stress has been demonstrated as a critical environmental trigger of the development of MDD. Acute stressful life events stimulate synthesizing and releasing of glucocorticoids from adrenal gland into blood circulation. The elevated glucocorticoids arrive in multiple tissues throughout all the brain including the hippocampus, hypothalamus, pituitary and amygdala, *et al*. Chronic glucocorticoids exposure exerts harmful effects on these tissues and then induces HPA axis hyperactivity, considered as an important molecular mechanism underling the pathology of MDD [2], [3].

The occurrence of HPA axis hyperactivity is a critical step in the pathological development of MDD [2], [3], [4]. Neurons secreting corticotrophin-releasing factor (CRF) in the paraventricular nucleus (PVN) of the hypothalamus play a key role in the response of the body to physiological stress [4]. In response to acute stress, neurons in the PVN synthesize and secrete CRF, which then stimulates the synthesis and release of adrenocorticotropic hormone (ACTH) from the anterior pituitary. ACTH then stimulates the synthesis and release of glucocorticoids from the adrenal cortex. This circuit is named HPA axis and its activity is negatively feedback regulated by the hippocampus and the hypothalamus itself when the excessive glucocorticoids reach these places [5], [6]. Under acute stressful condition, the negative feedback mechanism maintains the homeostasis of HPA axis activity. However, converging evidence shows that the balance in most depressive patients is broken, which is due to the disruption of the negative feedback mechanism after chronic stress exposure [7], [8]. Chronic stress-induced hyperactivity of HPA axis results in persistently elevated glucocorticoids level in the whole brain and finally lead to the onset of depressive behaviors [9]. Why chronic glucocorticoids exposure disrupt the negative feedback modulation of HPA axis but acute glucocorticoids exposure onset it and, glucocorticoids in which part of the brain account for chronic stress-induced HPA axis hyperactivity remain not well understood.

Glucocorticoids have been considered as a potential cause of stress-induced depression [10]. Glucocorticoids receptor (GR) and MR are the two main receptors mediating the stressful effect of glucocorticoids [11]. Decreased level of GR in the hippocampus has been found as the primary etiology of HPA axis hyperactivity in depression [11]. However, there is no direct evidence demonstrating that glucocorticoids account for chronic stress-induced depressive behaviors and the hyperactivity of HPA axis. Metyrapone, a synthetic steroidogenesis inhibitor, inhibits the synthesis of corticosteroids (glucocorticoids in rodents, CORT) by blocking the function of 11-β-hydroxylase, the enzyme responsible for converting deoxycorticosterone to CORT within the adrenal cortex [12]. Metyrapone is used to inhibit the synthesis of CORT under stressful state and attenuate the stressful effect [13]. In the present study, we used metyrapone (s.c., 100 mg/kg/d) in combination with chronic mild stress (CMS) model of depression to investigate whether glucocorticoids account for chronic stress-induced depressive-like behavior and HPA axis hyperactivity.

nNOS is the main synthesis enzyme of NO in the hippocampus [14]. Previously, our research identified a novel pathway, MR-nNOS pathway, in the hippocampus mediated the stress-induced depressive behaviors. Glucocorticoids up-regulate nNOS expression which then synthesizes excessive NO [15]. NO regulates the function of gene, lipid and protein by soluble guanlylyl cyclase-cyclic guanosine monophosphate (sGC-cGMP) pathway [16]. Furthermore, NO react with superoxide O_2_^−^ radical to generate peroxynitrite (ONOO–), which also can regulate the function of several molecules [17]. Intrahippocampal excessive NO disrupts the function of GR, which is proved as a key molecule mediating the negative feedback modulation of HPA axis, by sGC-cGMP and ONOO– pathway. Here, we investigated the exact roles of glucocorticoids in the hippocampus and hypothalamus in the development of the pathology of stress-induced depression and whether the proved pathway exists in both tissues.

By using metyrapone, our results demonstrated that chronic stress-induced persistent glucocorticoids elevation was required for chronic stress-induced hyperactivity of HPA axis and depressive-like behavior. More importantly, we found that the chronic exposure of glucocorticoids in the hippocampus led to the disruption of the feedback modulation mechanism of the HPA axis and depressive-like behavior, but the chronic exposure of glucocorticoids in the hypothalamus did not induce HPA axis hyperactivity and depressive-like behavior. The MR-nNOS-NO pathway mediated the different roles of glucocorticoids in the hippocampus and hypothalamus. To our knowledge, this is the first time to suggest only the negative feedback regulation of HPA axis by the hippocampus is impaired and account for the hyperactivity of HPA axis while the negative feedback regulation of HPA axis by the hypothalamus is not changed. Although the acute exposure of glucocorticoids both in the hippocampus and hypothalamus onset the negative feedback regulation of HPA axis, the chronic glucocorticoids elevation in the hypothalamus can't disrupt the negative feedback regulation owing to the lack of MR expression. This research reveals how glucocorticoids in different places in the brain are implicated in depression and would shed light on the recognition of the not-fully-understand role of the hypothalamus in the stress-induced pathology of depression.

## Materials and Methods

### Animals

Young adult (6- to 8-week-old) male ICR mice, young adult (6- to 7-week-old) male homozygous nNOS-deficient mice (B6; 129S4-Nos1tm1Plh, KO, stock number: 002633) and their wild-type controls of similar genetic background (B6129SF2, WT) (both from Jackson Laboratories; maintained at Model Animal Research Center of Nanjing University, Nanjing, China), were used in this study. Animals were housed in an air-conditioned room (20±2°C), with a 12-hour light–dark schedule food and water *ad libitum*, except when specified otherwise. The light begins at 7 AM every day. Animal maintenance and use procedures were in accordance with the NIH Guide for Care and Use of Laboratory Animals and approved by the Institutional Animal Care and Use Committee of Nanjing Medical University.

72 male ICR mice were used to investigated whether glucocorticoids is required for chronic stress-induced depression. Among them, 19 male ICR mice were used to measure the concentration of CORT, 40 male ICR mice were used to perform behavior tests, and 12 male ICR mice were used to perform western blotting. 26 male ICR mice were used to investigate the effect of metyrapone on depressive-like behavior. And 8 male ICR mice were used to investigate the effect of metyrapone on CRF expression. To test whether high concentration of glucocorticoids is sufficient to induce depressive behaviors and hyperactivity of HPA axis, 28 male ICR mice were used for behavior tests and 10 male ICR mice were used for western blotting. 22 male ICR mice were used for testing the effect of DMSO on behavior tests. 78 male ICR mice were used to investigate the effect of CORT in the hypothalamus (40 for behavior tests; 32 for western blotting; 14 for Elisa). 68 male ICR mice were used to investigate the effect of CORT in the hippocampus (6 for western blotting; 20 for Elisa; 6 for PCR; 6 for immunofluorescence staining; 30 for behavior tests). In the MR-nNOS part, 18 male ICR mice were used measure CORT level, 6 mice were used for immunofluorescence staining, and 79 mice were used for western blotting. To investigate the role of NO, 9 male ICR mice were used to do nNOS immunofluorescence staining, 9 mice were used to measure NO concentration, and 16 male ICR mice were used to do western blotting. In addition, 8 nNOS-deficient mice plus 8 wild-type were used for performing western blotting and 8 nNOS-deficient mice plus 8 wild-type were used for performing immunofluorescence staining.

### Drugs

CORT (40 mg/kg/d), dimethyl sulfoxide (DMSO, 20 mg/ml, 250 µl/kg/d) and metyrapone (100 mg/kg/d) were subcutaneously injected for 28 days. CORT and metyrapone were purchased from Sigma. CORT was diluted in DMSO. Metyrapone was diluted in sterile physiological saline. 20 µM CPTIO, 10 µM ODQ, or 10 mM DETA/NONOate was infused into the DG regions of the hippocampus or into the PVN regions of the hypothalamus. All these drugs were purchased from Sigma-Aldrich.

### CMS procedure and depressive-like behavior measurement

The procedure of CMS was designed as described previously [14]. We prolonged the CMS duration to 4 weeks. The stress factors in the first week were applied in the fourth week repeatedly. Stress-induced modifications in mice were assessed using body weight gain, immobility time in the tail suspension test (TST) and forced swimming test (FST), open-field test (OFT), and sucrose preference test (SPT). The TST, FST, and OFT were measured as described previously [14], [15]. The duration of immobility in the TST and FST was recorded using the Hamilton kinder TS100 on PC computer and Motor- Monitor System SF16R, respectively. We calculated the immobility time during the last 4 minutes as valid immobility time in the FST. The exclude criteria for TST analysis include the dropping of the mice from the tail hook and the climbing of the mice up the tail hook. The exclude criteria for FST analysis include the disability of the mice to swim and the mice floating all the time.

For the OFT, the test arena was constructed of a plastic plate (56.13–56.13 cm); lines drawn on the plate divided it into 256 squares. It was surrounded by a 35.18-cm-high plastic wall. Each mouse was placed on a corner square of the arena, facing the corner, and allowed to freely explore the open field for 5 min per trial. After each trial, the plate was cleaned with 70% EtOH. No stressor was applied to the animals for at least 12 h before the test. Mobility was scored when an animal crossed a sector border with both its hind limbs. Parameters assessed were the number of square crossings (horizontal) and the times of standing (vertical) during the 5-min test.

The SPT was performed as described previously [18]. In brief, an SPT consisted of first removing the food and water from each mouse's cage for a period of 20 h. Water and 1% sucrose were then placed in the cages in pre-weighed plastic bottles and animals were allowed to consume the fluids for a period of 10 h. The bottles were then removed and weighed. Two baseline fluid intake tests were performed, separated by at least 5 d, and the results were averaged. Fluid intake was calculated on an absolute basis (sucrose and water intake separately) and as a preference score (sucrose preference, relative to total fluid intake). A preference score is calculated as the ratio of the intake of 1% sucrose water relative to total fluid intake including 1% sucrose water and blank water during 10 hours after 20 h food and water deprivation. The exclude criteria for SPT analysis include the leaking of the bottles.

### Western blotting

Western blotting analysis of samples from hippocampal or hypothalamic tissues of animals was performed as described previously [14]. The primary antibodies were as follows: mouse anti-GAPDH (1∶4000; Santa Cruz Biotechnology), rabbit anti-nNOS (1∶1000; Millipore Bioscience Research Reagents), rabbit anti-GR (1∶200; Santa Cruz Biotechnology), rabbit anti-MR (1∶3000; Santa Cruz Biotechnology), mouse anti-nitrotyrosine (1∶3000; Millipore Bioscience Research Reagents), rabbit anti-β-actin antibody (1∶1000; Sigma-Aldrich) and rabbit anti-corticotrophin-releasing factor (anti-CRF; 1∶500; Santa Cruz Biotechnology). Appropriate horseradish peroxidase-linked secondary antibodies were used for detection by enhanced chemiluminescence (Pierce).

### RNA extraction and reverse transcription-PCR

Total RNA was extracted from the hippocampus using Trizol reagent according to the manufacturer's instructions (Sigma). The primers for mouse CRF, and mouse GAPDH were as follows: for CRF (139 bp): forward, 5′-cctcagccggttctgatcc-3′; reverse, 5′-gcggaaaaagttagccgcag-3′. For GAPDH (550 bp): forward, 5′-aggccggtgctgagtatgtc-3′; reverse, 5′- tgcctgcttcaccaccttct-3′. PCR conditions were 30 cycles of denaturation at 94 °C for 45 s, annealing at 55 °C for 45 s, and extension at 72°C for 45 sec. PCR products were separated by electrophoresis through 1.5% agarose gel containing 0.5% 1 g/mL ethidium bromide and imaged using a BioDoc-IT imaging system (Bio-Rad); band intensities were determined using GS-710 calibrated imaging Densitometer (Bio-Rad). The mRNA for GAPDH was included in the PCR mixture as a standard.

### Immunohistochemistry

The nNOS-positive cells were counted in a one-in-six series of sections (240 µm apart) throughout the rostrocaudal extent of the granule cell layer based on the pictures taken through a ×10 objective (Leica). To determine the total number of nNOS-positive cells per dentate gyrus, the counts from sampled sections were averaged and the mean values were multiplied by the total number of sections. The total numbers of CRF-positive neurons in the PVN were calculated in a one-in-three series of sections (120 µm apart) based on the pictures taken through a ×10 objective. The total number of CRF-positive cells per PVN was averaged and the mean values were multiplied by the total number of sections. We randomly chose 10 CRF-positive neurons in this area to measure brightness of staining. The mean values of florescence intensity were analyzed by ImagePro (Media Cybernetics).

The primary antibodies used for immunofluorescence staining were as follows: rabbit anti-nNOS (1∶200; abcam), rabbit anti-CRF (1∶200; Santa Cruz Biotechnology). The fluorescent secondary antibodies used were affinity-purified goat anti-rabbit Cy3 (1∶400; Jackson immunoresearch), goat anti-rabbit 488 (1∶400; Jackson immunoresearch).

### Corticosterone level measurement

For basal CORT level measurement, mice were decapitated between 9:00 and 10:00 AM. Blood from angulus oculi vessels was collected in heparinized tubes, and corticosterone in plasma was measured with a corticosterone ELISA Kit according to the instructions of the manufacturer (Cayman Chemical Company, USA) [15]. For the concentration of CORT in hypothalamus measurement, the whole hypothalamus were dissected between 9:00 and 10:00 AM and homogenized in 200 µl physiological saline (mixed liquor). CORT levels in the mixed liquor were measured by ELISA. The concentration of CORT in the hypothalamus was calculated as the concentration in mixed liquor multiplied by the ratio of the weight of the mixed liquor to the weight of hypothalamus. The detection limit of this kit is approximately 40 pg/ml.

### NO concentration measurement

NO contents from the hippocampus or hypothalamus were determined as previously [14]. The samples were weighed, homogenized in 10 volumes of deionized water and centrifuged at 1000 g for 15 min at 4°C. NOx content was measured in supernatants using a commercially available kit (Jiancheng Bioengineering Co., Nanjing, China) and expressed as nanomole/mg protein.

### Stereotaxic surgery

Stereotaxic surgery was used to deliver drugs into the PVN of the hypothalamus (1 µl, coordinates: 0.85 mm caudal to bregma, 0.15 mm lateral to the midline, and 5.5 mm below the surface of the skull) [19] or into the DG of the hippocampus (2 µl, coordinates: 2.3 mm posterior to bregma, 1.3 mm lateral to the midline, and 2.0 mm below dura). Adult mice were anesthetized with 0.07 ml of a mixture of ketamine (90.9 mg/ml) and xylazine (9.1 mg/ml) and placed in a stereotaxic apparatus. A volume of 2 µl CORT, CPTIO, ODQ, DETA/NONOate or DMSO was infused into bilateral DG in different experiments. And, a volume of 1 ul CORT, DETA/NONOate or DMSO was infused into bilateral PVN in different experiments. All the infusion rates were 0.25 µl/min.

### Statistical analyses

Comparisons among multiple groups were made with one-way ANOVA followed by Scheffe's *post hoc* test. A 2-way ANOVA was used to assess possible differences of some molecule expression which were affected by drugs or stress between different positions (hippocampus and hypothalamus). Comparisons between two groups were made with the two-tailed Student's *t* test. Data were presented as mean ± SEM; p<0.05 was considered statistically significant.

## Results

### Chronic stress-induced depressive-like behavior and HPA axis hyperactivity requires glucocorticoids

**Figure 1:**
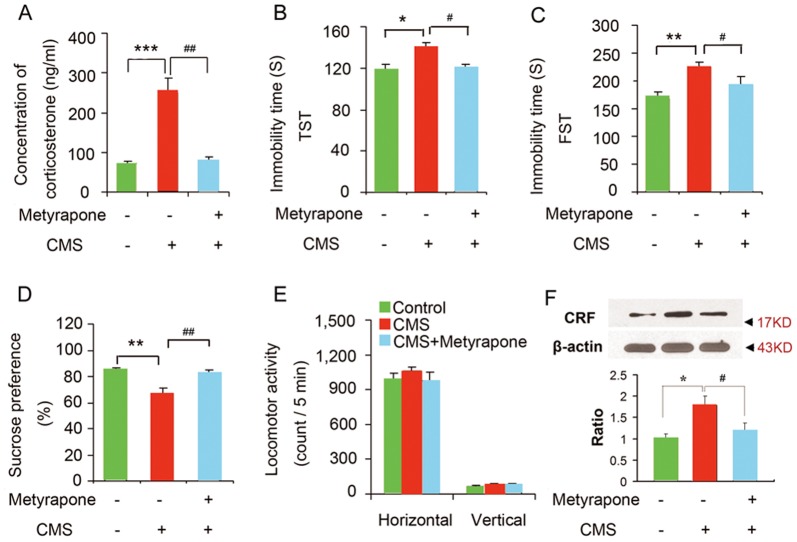
Chronic stress induced-depression requires glucocorticoids. (A) Plasma CORT levels in the mice exposed to CMS with or without metyrapone (s.c., 100 mg/kg/d) for 28 days (control n = 7, CMS n = 6, CMS+ metyrapone n = 6). (B–E) Metyrapone abolished CMS-induced behavioral modifications. Immobility time in the TST (B, control n = 12, CMS n = 12, CMS+ metyrapone n = 16) and FST (C, control n = 12, CMS n = 12, CMS+ metyrapone n = 16), sucrose preference (D, control n = 12, CMS n = 12, CMS+ metyrapone n = 15), and locomotor activity (E, control n = 12, CMS n = 12, CMS+ metyrapone n = 16) of the mice exposed to CMS with or without metyrapone (s.c., 100 mg/kg/d) for 28 days. (F) Western blotting showing CRF levels in the hypothalamus of adult mice exposed to CMS with or without metyrapone (n = 4 for each group), arrow heads indicate the location of nearest band in the ladder. Error bars denote SEM, * p<0.05, ** p<0.01, *** p<0.001, compared to control; # p<0.05, ## p<0.01, ### p<0.001, compared to CMS; one-way ANOVA.

Stressful events have been considered as the environmental cause of depression [1], [2], [3]. Chronic stress induces many morphological changes of neurons and functional abnormality of molecules related to depression. Although the persistent increase of glucocorticoids has been considered as a primary factor of these changes, there is no direct evidence demonstrating glucocorticoids account for chronic stress-induced depressive behaviors and hyperactivity of HPA axis. To explore this question, we used the CMS as the model depression and measured CORT levels in the plasma and evaluated despair behaviors by the tail suspension test (TST), and forced swimming test (FST) and sensitivity to reward by sucrose preference tests (SPT), which are commonly used to estimate the depressive behavior in rodents. The mice exposed to CMS for 28 days exhibited a significant increase in CORT level and prolonged immobility time in the TST and FST, and a significant decrease in sucrose preference (Figure 1). During CMS exposure, treatment with metyrapone (s.c., 100 mg/kg/d, for 28 days), a type of corticosteroid synthesis inhibitor, blocked the CMS-induced CORT level elevation (F_(2, 16)_ = 7.47; p = 0.001 control VS CMS; P = 0.01, CMS VS CMS+ metyrapone; one-way ANOVA, Figure 1A), immobility time prolongation (TST: F_(2, 37)_ = 5.36; Figure 1B; p = 0.016 control VS CMS; P = 0.041, CMS VS CMS + metyrapone, one-way ANOVA, Figure 1B; FST: F_(2, 37)_ = 7.75; p = 0.003 control VS CMS; P = 0.013, CMS VS CMS + metyrapone, one-way ANOVA, Figure 1C), and sucrose preference decrease (F_(2, 36)_ = 6.62; p = 0.007, control VS CMS; P = 0.018, CMS VS CMS+ metyrapone; one-way ANOVA, Figure 1D). The treatments above did not change the locomotor activity of mice (Figure 1E). Furthermore, we observed that the CRF expression was significantly increased in the hypothalamus of mice exposed to CMS for 28 days, reversed by metyrapone treatment (F_(2, 9)_ = 9.92; p = 0.007, control VS CMS; P = 0.031, CMS VS CMS + metyrapone; one-way ANOVA, Figure 1F). To investigate the effect of metyrapone on behaviors and CRF expression, we treated mice with metyrapone (s.c., 100 mg/kg/d, for 28 days). Depressive-like behavior tests showed that metyrapone did not change the immobility time in TST (immobility time: control 113.307±12.431, metyrapone 121.289±22.580, p = 0.947, t = 0.106, n = 13 each group, two-tailed Student's *t* test) and FST (immobility time: control 177.210±19.082, metyrapone 170.001±15.527, p = 0.958, t = 0.101, n = 13 each group, two-tailed Student's *t* test) compared to control. Consistently, metyrapone did not change sucrose preference of mice (control 87.902%±5.118%, metyrapone 86.349%±7.524%, p = 0.981, n = 13 each group, t = 0.058, two-tailed Student's *t* test). Also, the treatment did not change the locomotor activity of mice (data not shown). In addition, metyrapone did not induce significant changes in CRF expression in the hypothalamus measured by western blotting (p = 0.249, t = 1.274, n = 4 for each group, two-tailed Student's *t* test). Thus, these data collectively indicates that glucocorticoids are responsible for the chronic stress-induced depressive-like behaviors and hyperactivity of HPA axis.

### Glucocorticoids is sufficient to induce depressive-like behavior and HPA axis hyperactivity

**Figure 2:**
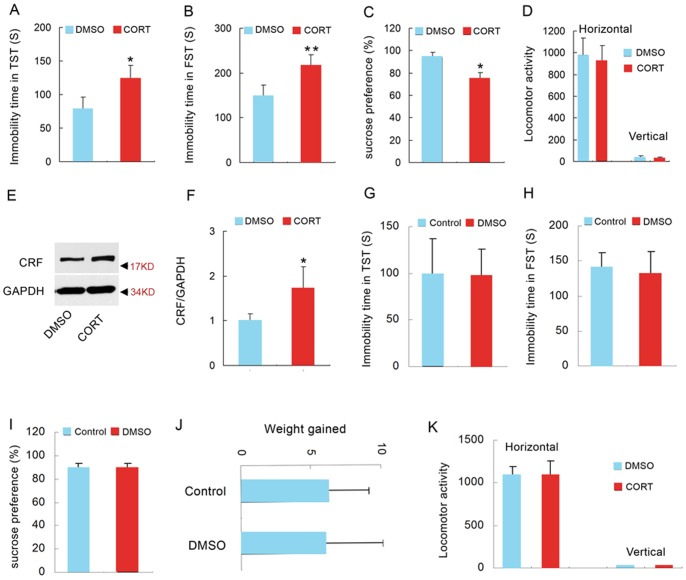
High concentration of glucocorticoids is sufficient to induce depressive behaviors and hyperactivity of HPA axis. (A) Immobility time in the TST after 28 days CORT treatment (40 mg/kg/d, s.c.) (n = 14 for each group). (B) Immobility time in the FST 28 days CORT treatment (40 mg/kg/d, s.c.) (n = 14 for each group). (C) Sucrose preference of mice 28 days CORT treatment (40 mg/kg/d, s.c.) (n = 14 for each group). (D) Locomotor activity of the mice 28 days CORT treatment (40 mg/kg/d, s.c.) (n = 14 for each group). (E) Representative western blotting of CRF and GAPDH in the hypothalamus of mice treated with CORT (40 mg/kg/d s.c.) or DMSO for 28 d (n = 5 for each group) and (F) the statistical data of the western blotting experiment. Arrow heads indicate the location of nearest band in the ladder. (G) Immobility time in the TST after 28 days treatment with DMSO (20 µl/g/d, s.c.) (n = 11 for each group). (H) Immobility time in the FST after 28 days treatment with DMSO (20 µl/g/d, s.c.) (n = 11 for each group). (I) Sucrose preference after 28 days treatment with DMSO (20 µl/g/d, s.c.) (n = 11 for each group). (J) Increased weight of mice after 28 days treatment with DMSO (20 µl/g/d, s.c.) (n = 11 for each group). (K) Locomotor activity of the mice after 28 days treatment with DMSO (20 µl/g/d, s.c.) (n = 11 for each group). Error bars denote SEM, *p<0.05, two-tailed Student's *t* test.

Metyrapone blocked the synthesis of glucocorticoids (Figure 1), but it also might affect other molecules. To further determine whether glucocorticoids *per se* can induce the pathology of depression, we administrated adult mice with CORT (40 mg/kg/d, s.c.) for 28 days and measured depressive-like behaviors by TST, FST, and SPT. The mice exposed to CORT for 28 days exhibited a significant increase in immobility time in the TST and FST (TST: p = 0.013, t = 2.775, two-tailed Student's *t* test, Figure 2A; FST: p = 0.002, t = 3.435, two-tailed Student's *t* test, Figure 2B), and a significant decrease in sucrose preference (94.190±4.255% for DMSO, 72.740±7.638% for CORT, p = 0.037, t = 2.329, two-tailed Student's *t* test, Figure 2C), indicating that mice exposed to CORT display depressive-like behaviors. In addition, the treatments did not change the locomotor activity of mice (p = 0.433 for horizontal, t = 0.701; p = 0.845 for vertical, t = 0.021; two-tailed Student's *t* test, Figure 2D). Meanwhile, we measured the expression of CRF in the hypothalamus by western blotting. The western blotting result showed an overexpression of CRF in the hypothalamus of mice exposed to CORT (p = 0.002, t = 4.501, two-tailed Student's *t* test, Figure 2E, F), indicating the hyperactivity of HPA axis. Because DMSO can be damaging to tissues, we assessed the effect of long term DMSO exposure (20 mg/ml, 250 µl/kg/d, 28 days) on depressive-like behavior. Compared to control group, long term DMSO exposure did not change behavior phenotypes of mice in TST (p = 0.746, t = 0.255, two-tailed Student's *t* test, Figure 2G), FST (p = 0.638, t = 0.503, two-tailed Student's *t* test, Figure 2H) and SPT (p = 0.755, t = 0.246, two-tailed Student's *t* test, Figure 2I), excluding the possibility that damaging effect of DMSO account for the stressful effect of CORT. And, there was no significant difference in the weight gained and the locomotor activity of mice between DMSO and control group (Weight gain: p = 0.514, t = 0.570, two-tailed Student's *t* test, Figure 2J; locomotor activity: p = 0.701 for horizontal, t = 0.293; p = 0.538 for vertical, t = 0.546; two-tailed Student's *t* test, Figure 2K). Thus, these data indicate that high concentration of glucocorticoids is sufficient to induce stress-related depressive-like behavior changes and the hyperactivity of HPA axis.

### The role of the hypothalamus in glucocorticoids-induced depressive-like behavior and HPA axis hyperactivity

**Figure 3:**
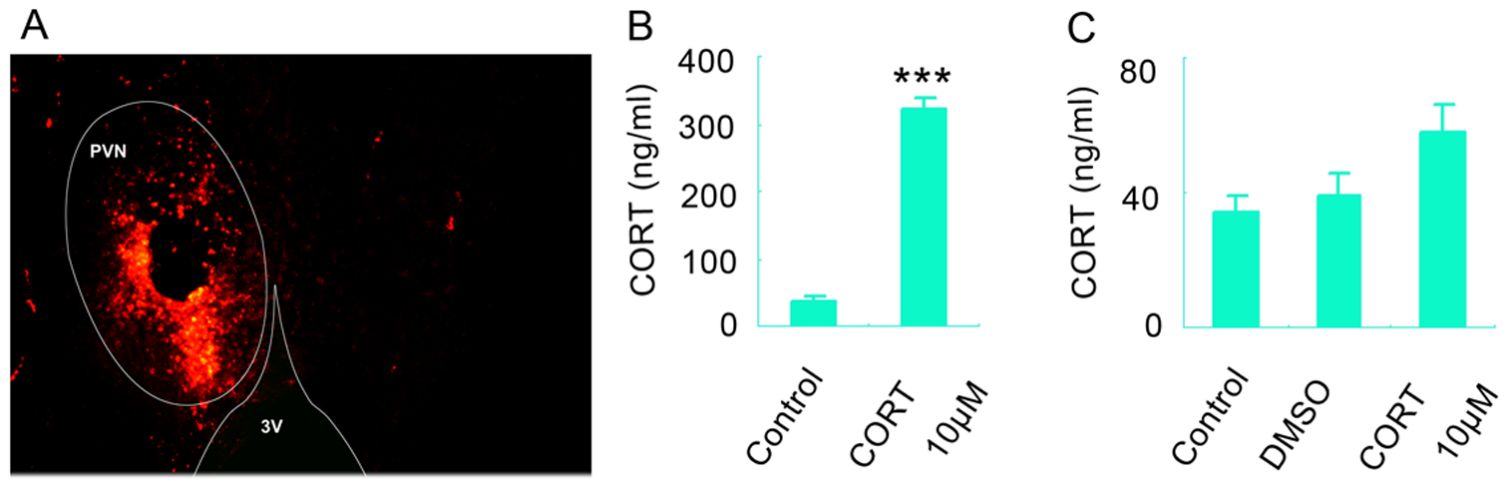
Selective infusion of CORT into the hypothalamus increases the level of CORT in the hypothalamus persistently. (A) Representative photo indicated the target of the infusion (the left PVN region of the hypothalamus). 3V: third ventricle. (B) The concentration of CORT in the whole hypothalamus 24 h after infusion of CORT. n = 3 for each group. (C) The concentration of CORT in the whole hypothalamus 28 d after infusion of CORT. n = 3 for each group. Error bars denote SEM, *** p<0.001 compared to control group, two-tailed Student's *t* test in (B), one-way ANOVA in (C).

**Figure 4:**
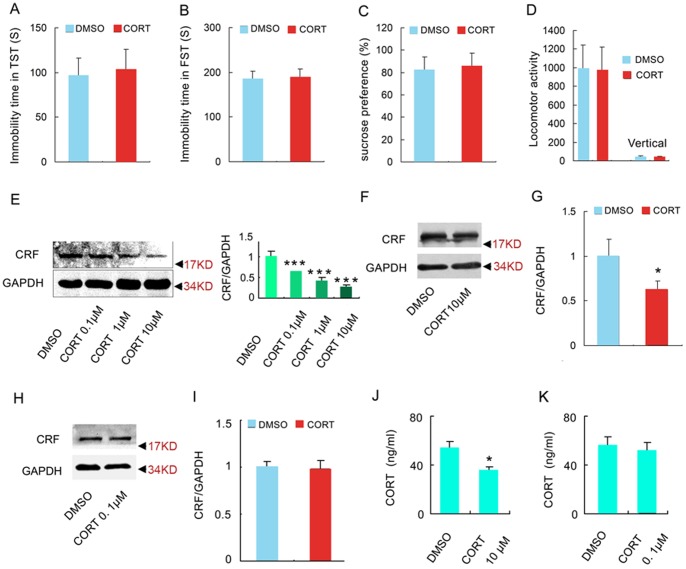
High concentration of glucocorticoids in the hypothalamus exerts negative regulation of HPA axis. Depressive-like behaviors test were measured 28 d after infusion of CORT (10 µM, 1 µl) into each PVN: (A) Immobility time in the TST (n = 15 for each group). (B) Immobility time in the FST (n = 15 for each group). (C) Sucrose preference of mice (n = 15 for each group). (D) Locomotor activity of the mice within 5 min (n = 15 for each group). (E) Representative western blotting of CRF and GAPDH in the hypothalamus (left) and the statistical data of the western blotting experiment (right) (n = 4 for each group). Representative western blotting of CRF and GAPDH in the hypothalamus 28days after the infusion of 10 µM CORT (F) or 0.1 µM CORT into the PVN (H) and their statistical data (G, I), n = 4 for each group. Arrow heads indicate the location of nearest band in the ladder. (J) The concentration of CORT in the plasma 28 d after infusion. n = 4 for each group, the 10 µM CORT group and the 0.1 µM CORT were analyzed separately. Error bars denote SEM, *p<0.05, ***p<0.001, two-tailed Student's *t* test except for one-way ANOVA in (E).

Our results showed that stress-induced glucocorticoids elevation was a critical primary environmental causative factor of depression. Then the question is which part of the brain actually underlies the stressful effect of systemic CORT increase? Whether the action of glucocorticoids in the hypothalamus contributes to the pathology of depression? To explore this possibility, we infused a volume of high concentration of CORT (10 µM, 1 µl) or DMSO (1 µl) into each PVN zone of the hypothalamus where was considered as the initial onset site of HPA axis activity [20]. To check the accuracy of the infusion, we infused a volume of 1 µl DyLight fluorescent dye Cy3 into the left PVN zone of the hypothalamus of mice and the brain slices were detected under fluorescence microscope 2 h later. It was shown that the red fluorescence was only observed in the left PVN zone but no other regions (Figure 3A). To further confirm the hypothalamic target of the infusion, we measured the concentration of CORT in the whole hypothalamus. The content of CORT in the hypothalamus was strikingly high at 24 h after infusion (Figure 3B, p = 0.001, t = 8.610, two-tailed Student's *t* test) compared to control group and remained slightly elevated at 28 days after infusion (F(2, 6) = 26.37; p = 0.057, one-way ANOVA, Figure 3C) compared to control group. In addition, DMSO infusion did not change the CORT concentration in the hypothalamus compared to control group at 28 days after infusion (F(2, 6) = 26.37; p = 0.485, one-way ANOVA, Figure 3C). The depressive-like behaviors of mice were measured by TST, FST and SPT at 28 days after infusion. Surprisingly, there was no significant difference in the immobility time in TST and FST (TST: 94.870±19.283 for DMSO, 102.374±25.236 for CORT, p = 0.521, t = 0.689, two-tailed Student's *t* test, Figure 4A; FST: 206.539±20.791 for DMSO, 204.583±25.210 for CORT, p = 0.834, t = 0.017, two-tailed Student's *t* test, Figure 4B) and no significant sucrose preference in SPT (81.230±12.975% for DMSO, 85.577±11.881% for CORT, p = 0.739, t = 0.073, two-tailed Student's *t* test, Figure 4C) between CORT and DMSO group. Additionally, the treatment did not change the locomotor activity of mice (p = 0.856 for horizontal, t = 0.103; p = 0.881 for vertical, t = 0.090; two-tailed Student's *t* test, Figure 4D). These results suggest that chronic exposure to CORT in the hypothalamus can't induce depressive-like behavior in mice.

Furthermore, we investigated the role of hypothalamus in the chronic glucocorticoids exposure-induced hyperactivity of HPA axis. We infused a volume of 1 µl CORT (0.1, 1, 10 µM, respectively) into bilateral PVN regions and measured the CRF expression in the hypothalamus 2 h later. The drug significantly decreased CRF expression in a concentration-dependent way (60.52%, 40.1%, and 25.67% for 0.1, 1.0, and 10 µM CORT, respectively, relative to DMSO, F(3,12) = 176.450, one-way ANOVA, Figure 4E), suggesting the acute action of CORT in the PVN is to negatively modulate HPA axis activity. More importantly, western blotting results showed that the infusion of 10 µM CORT into the PVN regions still repressed the expression of CRF at 28days after the infusion (p = 0.009, t = 3.726, two-tailed Student's *t* test, Figure 4F, G), but which was not observed in 0.1 µM CORT group (p = 0.739, t = 0.427, two-tailed Student's t test, Figure 4H, I). Consistently, microinjection of a volume of 1 µl of 10 µM CORT but not 0.1 µM CORT into bilateral PVN regions caused a significant decrease in CORT level in the plasma at 28days after the microinjection (For 10 µM CORT: p = 0.007, t = 3.980, two-tailed Student's *t* test, Figure 4J; For 0.1 µM CORT: p = 0.518, t = 0.695, two-tailed Student's *t* test, Figure 4K). Therefore, a persistent elevation of CORT in the PVN regions also exerted an inhibitory effect on HPA axis activity, which was in inverse to the result of systemically treatment with CORT experiments (Figure 2E, F). Altogether, these results could imply that chronic stress-related hyperactivity of HPA axis and depressive behavior does not attribute to the action of glucocorticoids in the PVN of the hypothalamus.

### The role of the hippocampus in glucocorticoids-induced depressive-like behavior and HPA axis hyperactivity

**Figure 5:**
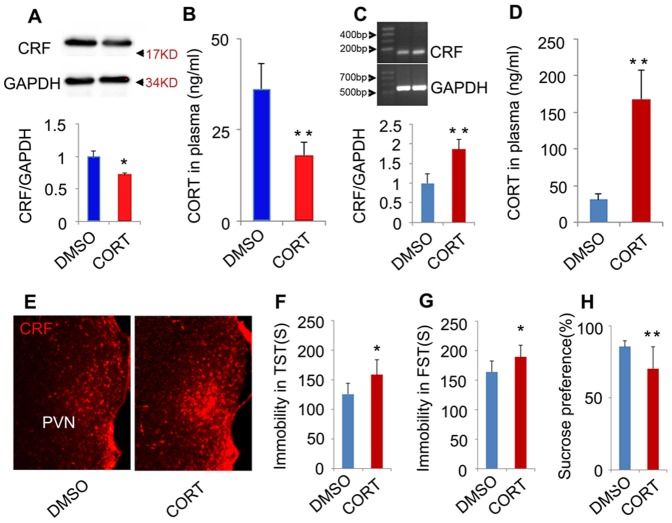
High concentration of glucocorticoids in the hippocampus plays opposite roles in acute and chronic phase in regulating HPA axis activity. (A) Western blotting showing CRF protein expression in the hypothalamus 2 hours after the infusion of CORT (10 µM, 2 µl) into the DG regions. n = 3 for each group. Arrow heads indicate the location of nearest band in the ladder. (B) The CORT level in the plasma 2 hours after the infusion of CORT (10 µM, 2 µl) into the DG regions. n = 4 for each group. (C) RT-PCR showing CRF mRNA in the hypothalamus 28 days after the infusion of CORT (10 µM, 2 µl) into the DG regions. n = 3 for each group. (D) The CORT level in the plasma 28 days after the infusion of CORT (10 µM, 2 µl) into the DG regions. n = 6 for each group. (E) Representative imaging of CRF-positive cells in the PVN of the hypothalamus 28 days after the infusion of CORT (10 µM, 2 µl) into the DG regions. The result was repeated in 3 mice for each group (data not shown). Immobility time in the TST (F, DMSO n = 12, CORT n = 16) and FST (G, DMSO n = 12, CORT = 16), sucrose preference (H, DMSO n = 12, CORT = 15) 28 days after the infusion of CORT (10 µM, 2 µl) into the DG regions. Error bars denote SEM, *P<0.05, **P<0.01, two-tailed Student's *t* test.

Next, we investigated the role of CORT in the hippocampus, the main negative feedback center of HPA axis. We infused a volume of high concentration of CORT (10 µM, 2 µl) or DMSO (2 µl) into the DG regions of bilateral hippocampi. Two hours after the infusion, there was a marked decrease in CRF expression in the hypothalamus (p = 0.012, t = 3.751, two-tailed Student's *t* test, Figure 5A) and in the CORT level in the plasma (p = 0.007, t = 5.208, two-tailed Student's *t* test, Figure 5B). On the contrary, 28 days later, RT-PCR data showed that the expression of CRF mRNA in the hypothalamus strikingly increased (p = 0.003, t = 7.173, two-tailed Student's *t* test, Figure 5C). In addition, the CORT level in the plasma increased up to nearly five times as high as the level in DMSO group (p = 0.003, t = 3.822, two-tailed Student's *t* test, Figure 5D). Immunohistochemistry experiment also certified that CRF content in the PVN was remarkably higher in CORT group compared to DMSO group (Figure 5E, data not shown). Consistently, 28 days after infusion, mice displayed a prolonged immobility time in the TST and FST (TST: p = 0.016, t = 2.693, two-tailed Student's t test, Figure 5F; FST: p = 0.015, t = 2.702, two-tailed Student's t test, Figure 5G), and a significant decrease in sucrose preference in SPT (p = 0.009, t = 2.904, two-tailed Student's *t* test, Figure 5H). Together, these data suggest that acute exposure to high concentration of glucocorticoids in the hippocampus exerts negative feedback regulation of HPA axis, but chronic exposure of glucocorticoids destroy the negative feedback regulation HPA axis by hippocampus and finally induces depressive behaviors and hyperactivity of HPA axis.

### MR-nNOS pathway mediates the different roles of glucocorticoids in the hippocampus and hypothalamus

**Figure 6:**
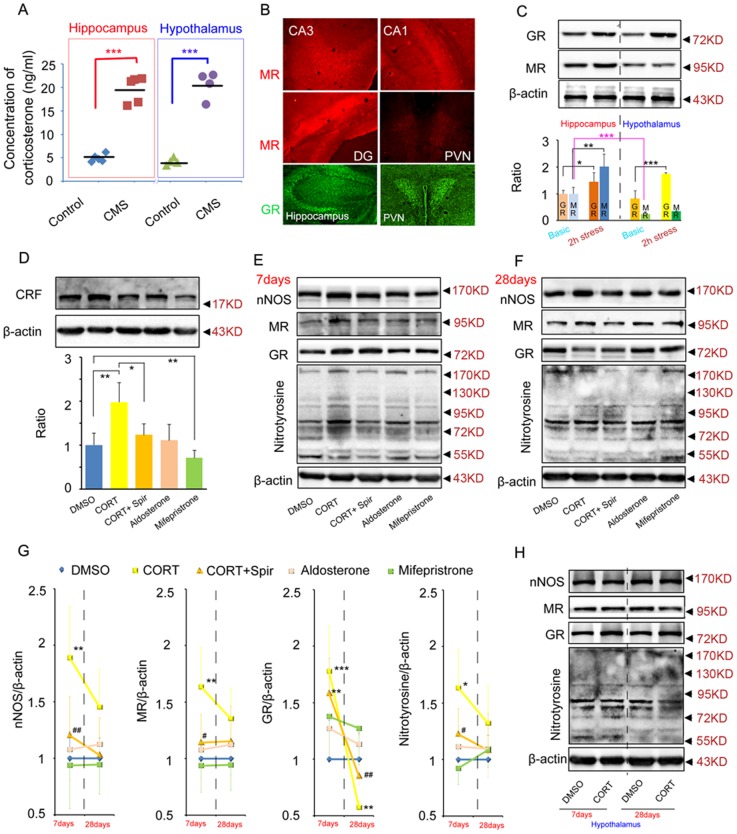
MR-nNOS pathway mediates the different roles of glucocorticoids in the hippocampus and hypothalamus in regulating HPA axis activity. (A) Hippocampal and hypothalamic CORT concentrations of adult mice under normal condition (control) or exposed to 28 days CMS. CMS led to a similar elevation of CORT concentration both in the hippocampus (n = 5 for each group) and hypothalamus (n = 4 for each group). (B) Representative photos of MR and GR immunofluorescence in the hippocampus and hypothalamus. The immunofluorescence was repeated at least in 3 mice. (C) Western blotting showing GR, MR protein expression in the hippocampus and hypothalamus under basic condition or under acute stressful condition. n = 3 for each group. (D) Western blotting showing CRF protein expression in the hypothalamus 28 days after microinjection of CORT with or without spironolactone, aldosterone, mifepristone into the DG. n = 4 for each group. (E–G) Western blotting showing nNOS, MR, GR, and nitrotyosine expression in the hippocampus 7 days (E) or 28 days (F) after infusion of CORT with or without spironolactone, aldosterone, mifepristone into the hippocampus. 7days: n = 4 for each group; 28days: n = 3 for each group. (H) Western blotting showing nNOS, MR, GR, and nitrotyrosine protein expression in the hypothalamus 7 days or 28 days after infusion of CORT into the hypothalamus. n = 3 for each group. Arrow heads indicate the location of nearest band in the ladder. Error bars denote SEM, *P<0.05, **P<0.01, ***P<0.001, compared to DMSO; ^#^P<0.05, ^##^P<0.05, compared to CORT. One-way ANOVA in (D, E, F, G), two-way ANOVA in (C, H).

Both in the hippocampus and hypothalamus, a short term elevation (2 h) of glucocorticoids exerted a negative regulation of HPA axis (Figure 4F–G, Figure 5A–B). A long term (28 d) elevation of glucocorticoids in hippocampus disrupted the negative feedback balance of HPA axis (Figure 5C–E). However, a long term (28 d) elevation of glucocorticoids in hypothalamus still exerted a negative regulation of HPA axis (Figure4H–I). These data suggested different roles of the hippocampus and hypothalamus in regulating HPA axis activity under chronic stress state. Moreover, the difference was not due to different levels of glucocorticoids in the hippocampus and hypothalamus under chronic stress state (28 days CMS exposure, in the hippocampus: 4.942±0.809 ng/ml in control, 19.615±1.327 ng/ml in CMS, p = 0.001, t = 5.041, two-tailed Student's *t* test; in the hypothalamus: 4.245±0.038 ng/ml in control, 20.598±1.446 ng/ml in CMS, p = 0.001, t = 5.959, two-tailed Student's *t* test; Figure 6A). To understand the molecular mechanism underling the different roles of glucocorticoids in the hippocampus and hypothalamus in regulating HPA axis activity, we compared GR and MR express pattern in the hippocampus and hypothalamus by immunofluorescence staining and western blotting. Immunofluorescence staining revealed that the expression level of MR in the hippocampus including CA1, CA3 and DG was markedly higher than the expression level of MR in hypothalamus including PVN (Figure 6B). But, there was no obvious difference in the GR expression levels between these two places (Figure 6B). We also measured the MR and GR protein expression in the hippocampus and hypothalamus under basic condition or under acute stressful condition (2 h restraint stress exposure). Western blotting results confirmed that MR protein level in the hypothalamus is significantly lower than the hippocampus under basic condition (F(3, 8) = 123.380, p = 0.001, two-way ANOVA, location × stress condition, Figure 6C) and that GR protein level was not observed significant difference between these two places under basic condition (F(3, 8) = 102.960, p = 0.418, two-way ANOVA, location × stress condition, Figure 6C). In addition, acute stress significantly increased MR level in the hippocampus (F(3, 8) = 123.380, p = 0.001, two-way ANOVA, location × stress condition, Figure 6C) but not in the hypothalamus (F(3, 8) = 123.380, p = 0.093, two-way ANOVA, location × stress condition, Figure 6C), implying the stress-induced MR expression might mediate the difference roles of the hippocampus and hypothalamus in regulating the HPA axis activity after chronic stress. To test this possibility, we designed to microinject CORT and pharmacological tool drugs into the hippocampus or hypothalamus to detect related molecules changes.

Firstly, to test the exact role of MR and GR in the hippocampus, we microinjected CORT (10 µM, 2 µl for each hippocampus, dissolved in DMSO) with or without spironolactone (MR antagonist, 10 µM, 2 µl), aldosterone (MR agonist, 10 µM, 2 µl), mifepristone (GR agonist, 10 µM, 2 µl) into the DG of the hippocampus (Figure 6D). 28 days after injection, we measured the CRF level in the hypothalamus and found that spironolactone blocked the CORT-induced CRF overexpression (F(4, 15) = 157.780, p = 0.001, one-way ANOVA, Figure 6D). However, aldosterone did not induce CRF overexpression compared to DMSO (F(4, 15) = 157.780, p = 0.726, one-way ANOVA, Figure 6D). Together, the results showed that glucocorticoids in the hippocampus regulated HPA axis by MR and MR activation in the hippocampus required glucocorticoids to impair the negative feedback regulation of HPA axis. In addition, selectively activation of GR by 28d mifepristone exposure in the hippocampus deceased hypothalamic CRF expression (F(4, 15) = 157.780, p = 0.007, one-way ANOVA, Figure 6D), implying the overexpression of GR in the hippocampus under acute stress (Figure 6C, D, E, G) did not participate in the CORT-induced pathology (such as impairment of the negative regulation of HPA axis) and that the role of GR in the hippocampus is to negatively regulate the activity of HPA axis.

To confirm the MR function and dissect the downstream molecular mechanism, we measured nNOS, MR, GR and nitrotyrosine in the hippocampus day 7 and day 28 after microinjection of CORT with or without spiristrone, aldosterone, and mifepristone into the DG of the hippocampus. At day 7, CORT, but not aldosterone, upregulated nNOS, MR, GR and nitrotyrosine level, and the upregualtion of nNOS, MR and nitrotyrosine but not GR were reversed by spironolactone (nNOS: F(4, 15) = 130.490, CORT VS DMSO p = 0.006, CORT+Spir VS CORT p = 0.009; MR: CORT VS DMSO p = 0.009, CORT+Spir VS CORT p = 0.014; GR: F(4, 15) = 157.480, CORT VS DMSO p = 0.001, CORT+Spir VS DMSO p = 0.005; nitrotyrosine: CORT VS DMSO p = 0.037, CORT+Spir VS CORT p = 0.039; One-way ANOVA, Figure 6E, G). At day 28, CORT induced a significant down-regulation of GR level in the hippocampus, which was reversed by spironolactone (GR: F(4, 10) = 97.040, CORT VS DMSO p = 0.001, CORT+Spir VS CORT p = 0.008, One-way ANOVA, Figure 6F, G). Meanwhile, hippocampal infusion of mifepristone did not affect the expression level of nNOS, MR, GR and nitrotyrosine in the hippocampus at both time points (Figure 6E, F, G).

Secondly, to test whether the pathway also exists in the hypothalamus, we microinjected CORT (1 µl, 10 µM, dissolved in DMSO) into bilateral PVN regions. Western blotting showed 7 days after infusion CORT induced an up-regulation of GR (F(3, 8) = 107.390, p = 0.187, two-way ANOVA, time x drug, Figure 6H, data not shown), which was similar to the situation in the hippocampus (Figure 6E, G). However, at day 28, CORT still induced GR up-regulation (F(3, 8) = 107.390, p = 0.019, two-way ANOVA, time x drug, Figure 6H, data not shown), which is extremely opposite to the situation in the hippocampus (Figure 6F, G). Importantly, CORT did not change nNOS, MR and nitrotyrosine expression at both time points in the hypothalamus (nNOS: F(3, 8) = 3.250, 7d: p = 0.505, 28d: p = 0.997, two-way ANOVA, time × drug; MR: F(3, 8) = 1.380, 7d: p = 0.597, 28d: p = 0.443, two-way ANOVA, time × drug; nitrotyrosine: F(3, 8) = 6.490, 7d: p = 0.348, 28d: p = 0.216, two-way ANOVA, time × drug,; Figure 6H).

Consistently, western blotting results showed that 28 days CMS exposure caused a significant decrease in GR expression in the hippocampus (Control: 1±0.128, CMS: 0.636±0.090, p = 0.016, t = 3.147, two-tailed Student's *t* test, n = 4 for each group) but not in the hypothalamus (Control: 1±0.170, CMS: 1.153±0.253, p = 0.525, t = 0.690, two-tailed Student's *t* test, n = 4 for each group). Thus, long term action of glucocorticoids in the hypothalamus did not disrupt the negative feedback regulation of HPA axis, which may be due to lacking the glucocorticoids-MR-nNOS- nitrotyrosine-GR pathway in the hypothalamus.

### NO mediates the different roles of glucocorticoids in the hippocampus and hypothalamus

**Figure 7:**
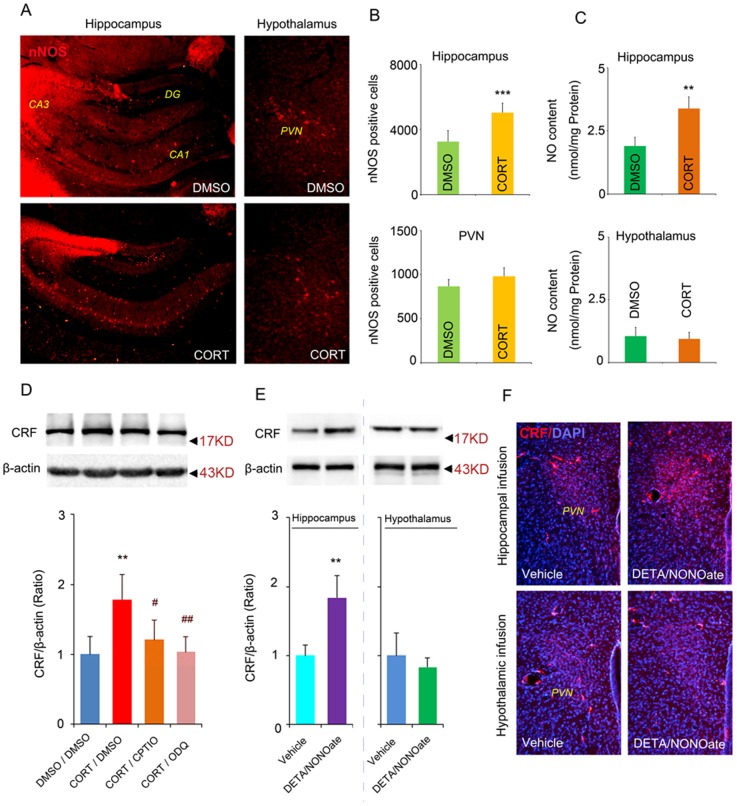
NO mediates the different roles of glucocorticoids in hippocampus and hypothalamus in regulating HPA axis activity. Representative imaging of nNOS-positive cells in the hippocampus (left column) and in the PVN regions of the hypothalamus (right column) after 28 days CORT (40 mg/kg, s.c.) or DMSO treatment (A) and the number of nNOS-positive cells (B), n = 4 in DMSO group, n = 5 in CORT group. (C) The concentration of NO in the hippocampus or hypothalamus after 28 days CORT treatment (40 mg/kg, s.c.), n = 4 in DMSO group, n = 5 in CORT group. (D) Western blotting showing CRF levels in the hypothalamus at day 28 of CORT treatment with or without CPTIO or ODQ, n = 4 for each group. CORT/DMSO represented administration of CORT via s.c. plus infusion of DMSO into bilateral DG of the hippocampus, and so on. (E) Western blotting showing CRF levels in the hypothalamus 28 days after infusion of DETA/NONOate, n = 4 for each group. Infusion of DETA/NONOate into the hippocampus increased CRF expression in the hypothalamus (left). Infusion of DETA/NONOate into the PVN regions of hypothalamus did not change the CRF expression in the hypothalamus (right). Arrow heads indicate the location of nearest band in the ladder. (F) Representative imaging of CRF-positive cells (red) and DAPI-labeled cells (blue) in the hypothalamus 28 days after infusion of DETA/NONOate into the hippocampus (upper row) or hypothalamus (bottom row). Note that the CRF signal in the PVN region of the hypothalamus in mice received DETA/NONOate infusion into the hippocampus was stronger. All imaging represent 4 individual mice. Error bars denote SEM, *P<0.05, **P<0.01, ***P<0.001 compared to control group, ^#^<0.05, ^##^P<0.01, compared to CORT/DMSO group, two-tailed Student's t test in (B, C, E), one-way ANOVA in (D).

Microinjection of CORT in the hippocampus but not in the hypothalamus increased nNOS protein level (Figure 6E, H). To confirm glucocorticoids specifically increase nNOS signal pathway under chronic stressful condition, we used CORT model of depression by treating mice with CORT for 28 days (40 mg/kg, s.c., dissolved in DMSO). Immunofluorescence staining demonstrated that the number of nNOS-positive cells increased all over the hippocampus, including CA1, CA3 and DG regions, in CORT group compared with DMSO group (3246.782±276.988 per hippocampus in DMSO group, 5027.484±262.740 per hippocampus in CORT group, p = 0.001, t = 5.408, two-tailed Student's *t* test, Figure 7A left, B). However, we observed no difference in the number of nNOS-positive cells in the PVN of the hypothalamus between CORT and DMSO group (863.491±128.322 per PVN in DMSO group, 978.384±129.677 per PVN in CORT group, p = 0.458, t = 0.736, two-tailed Student's *t* test, Figure 7A right, B). Consistently, NO concentration in the hippocampus but not in the hypothalamus was significantly increased in CORT-treated mice compared with DMSO-treated mice (Hippocampus:1.898±0.302 nmol/mg protein in DMSO group, 3.380±0.416 nmol/mg protein in CORT group, p = 0.003, t = 3.518, two-tailed Student's *t* test; Hypothalamus: 1.049±0.346 nmol/mg protein in DMSO group, 0.938±0.264 nmol/mg protein in CORT group, p = 0.785, t = 0.309, two-tailed Student's *t* test, Figure 7C). To further link hippocampal NO signal and the regulation of HPA axis by glucocorticoids, we treated mice with CORT (40 mg/kg, s.c., dissolved in DMSO) in combine with delivering CPTIO (a cell-impermeable NO scavenger, 2 µl, 20 µM/L) or ODQ (a soluble guanylate cyclase inhibitor, 2 µl, 10 µM/L) into bilateral hippocampi by microinjection at day 4. Both CPTIO and ODQ reduced the CORT-induced CRF-overexpression in the hypothalamus at day 28 (F(3,12) = 89.470, CORT/DMSO VS DMSO/DMSO p = 0.002, CORT/CPTIO VS CORT/DMSO p = 0.014, CORT/ODQ VS CORT/DMSO p = 0.001, One-way ANOVA, Figure 7D). To test whether the NO function depends on the nNOS, we infused DETA/NONOate, a NO donor [15], into bilateral DG regions of the hippocampus or bilateral PVN regions of the hypothalamus in nNOS knockout mice. More interestingly, western blotting showed that infusion of 100 µM DETA/NONOate into bilateral DG regions of the hippocampus (2 µl) but not into bilateral PVN regions of the hypothalamus (1 ul) induced an overexpression of CRF in the PVN regions (Hippocampal injection: p = 0.001, two-tailed Student's t test; Hypothalamic injection: p = 0.628, t = 0.549, two-tailed Student's t test, Figure 7E) 28 days after infusion, suggesting that hippocampal nNOS regulated HPA axis through NO pathway. Consistently, immunofluorescence staining showed similar changes (Figure 7F, data not shown). Together, these data imply that the difference in NO concentration mediates the different roles of glucocorticoids in the hippocampus and hypothalamus in regulating HPA axis activity.

## Discussion

**Figure 8:**
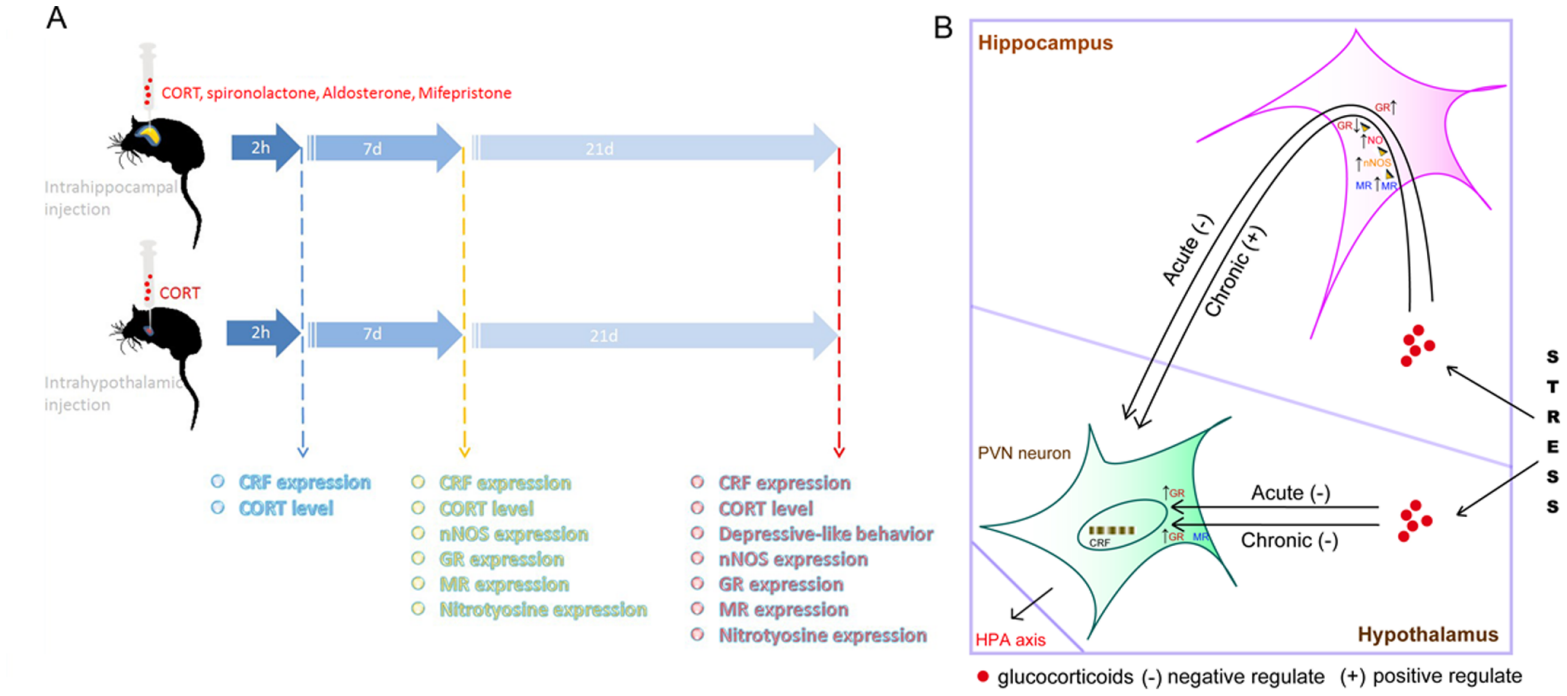
A model showing glucocorticoids plays different roles in the hippocampus and hypothalamus in modulating HPA axis activity under stressful state. (A) An overview of the design to compare the roles of glucocorticoids in the hippocampus and hypothalamus in HPA axis hyperactivity and depressive-like behaviors. (B) A model describing the different roles of glucocorticoids in the hippocampus and hypothalamus in depression. The Acute stress stimulates glucocorticoids synthesis and releasing in adrenal cortex. Under acute stressful state, when glucocorticoids arriving in the hippocampus or hypothalamus, glucocorticoids exerts negative regulation of the synthesis of CRF in PVN neurons by GR. Under chronic stressful state, glucocorticoids in the hippocampus impair the negative feedback modulation of the synthesis of CRF in PVN neurons in the hypothalamus by disrupting hippocampal GR. However, glucocorticoids in the hypothalamus still exert negative regulation on the synthesis of CRF in PVN neurons. Glucocorticoids in the hippocampus disrupt GR function through MR-nNOS-NO pathway. (−) exerting negative regulation of HPA axis activity; (+) exerting positive regulation of HPA axis activity.

It has been widely demonstrated that the synthesis and secretion of CRF in the PVN of the hypothalamus triggers the HPA axis activity [9]. The HPA axis is hyperactive in most patients with MDD, probably as a result of a primary over-synthesis of CRF [2], [8], [9], [11]. However, whether hypothalamus is implicated in the hyperactivity of HPA axis and depressive behaviors has not been investigated. The present study provides evidence that glucocorticoids is responsible for the chronic stress-induced depressive behaviors and hyperactivity of HPA axis and high concentration of glucocorticoids is sufficient to induce depressive behaviors and hyperactivity of HPA axis. More importantly, the acute action of glucocorticoids in both the hippocampus and hypothalamus contribute to the negative feedback regulation of the HPA axis. And, the chronic action of glucocorticoids in the hippocampus contributes to stress-induced hyperactivity of HPA axis and depressive behaviors. But, the chronic action of glucocorticoids in the hypothalamus still is to negatively modulate the activity of HPA axis. The original causation of the difference is the different contents of MR in the hippocampus and hypothalamus. Glucocorticoids activate the MR-nNOS-NO pathway and then results in the disruption of GR expression in the hippocampus, finally inducing HPA axis hyperactivity (Figure 8B). Due to the low level of MR in the hypothalamus, glucocorticoids in the hypothalamus dose not activate the MR-nNOS-NO pathway (Figure 8B). So the role of glucocorticoids in the hypothalamus after chronic stress exposure still is to exert negative regulation of HPA axis activity (Figure 8B). We reveal the different roles of the hippocampus and hypothalamus and its mechanism in regulating HPA axis activity and depressive behaviors (Figure 8).

In accordance with previous reports, our research demonstrated that long term exposure to high concentration of glucocorticoids resulted in depressive-like behaviors and hyperactivity of HPA axis in mice [21], [22]. Specifically, our research clarified long term glucocorticoids exposure accounted for and was sufficient to induce chronic stress-related depressive behaviors and the hyperactivity of HPA axis. Moreover, we demonstrated here that hypothalamic glucocorticoids were not involved in the stressful effects of chronic stress. And we reported here for the first time that the action of glucocorticoids in the hypothalamus still exerted negative feedback regulation of HPA axis after chronic stress. There are several endogenous inhibitory places of hypothalamus function including hippocampus, frontal cortex and hypothalamus self [9], [23]. Stress-induced glucocorticoids arrive at these tissues and exert negative regulation of the activity of HPA axis through GR [24]. But, whether the roles of these places in the pathology of HPA axis hyperactivity are the same remain unknown. We found that the expression level and the response to acute stress of GR were similar in the hippocampus and hypothalamus, which explain why acute exposure to glucocorticoids in the hippocampus and hypothalamus exerted negative feedback modulation of HPA axis similarly. We also found that reduced GR expression was only observed in the hippocampus but not in the hypothalamus, explaining why chronic exposure to glucocorticoids in the hippocampus but not in the hypothalamus induced HPA axis hyperactivity. In addition, different levels of MR in the hippocampus and hypothalamus (the level of MR in the hippocampus was much higher than the hypothalamus) was observed. More importantly, stress only unregulated MR expression in the hippocampus but not in the hypothalamus, which explained why glucocorticoids in the hippocampus but not in the hypothalamus activated MR-nNOS-NO-GR pathway. However, the reasons why stress only unregulated MR expression in the hippocampus but not in the hypothalamus need further research.

The inhibitory feedback regulation of HPA axis is disrupted in most depressive patients [2], [7]. In consistent with our previous study, nNOS produced NO in the hippocampus was crucial in chronic stress or glucocorticoids induced hyperactivity of HPA axis and depressive behavior [11], [12]. Extensive evidence demonstrate that nNOS produced NO negatively regulates hippocampal neurogenesis [24], [25], [26]. Numerous data show that hippocampal neurogenesis is required for the behavioral effects of antidepressants and the modulation of HPA axis [27], [28], [29]. Therefore, hippocampal new born neurons may mediate the effect of glucocorticoids-MR-nNOS-NO-GR pathway in the modulation of HPA axis by hippocampus. Although it was found the existence of neurogenesis in the adult hypothalamus, the function of the new born neurons was demonstrated as a regulator of feeding [30]. There is no evidence supporting the correlation between hypothalamic new born neurons and depression until now. Moreover, there is low level of MR expression (compared to the hippocampus) and no reaction in the MR expression in response to stress in the hypothalamus. Hence, the mechanism of modulation of the HPA axis by hypothalamus itself may be totally different with the hippocampus. However, considering beneficial effect of glucocorticoids in the hypothalamus on HPA axis homeostasis, it may be that the chronic stressful effect of glucocorticoids in the brain depends on the balance of the hippocampus and hypothalamus (Figure 8).

Considering that GR was thought as the key molecule mediating the negative feedback modulation of HPA axis [10], the similar GR level may explain why glucocorticoids in both hippocampus and hypothalamus (Figure 6) exert similar negative feedback regulation of HPA axis under normal state. More importantly, acute 2 h restraint stress increased GR expression both in the hippocampus and hypothalamus, confirming both the hippocampus and hypothalamus participate in the negative feedback regulation of HPA axis in response to acute stress. These data are consistent with our previous study that glucocorticoids-MR-nNOS-nitrotyrosine-GR pathway in the hippocampus mediate chronic stress-induced hyperactivity of HPA axis [15].

The half-life of glucocorticoids is very short. The measure of CORT concentration in the whole hypothalamus 24 h and 28 days after the infusion of high concentration of CORT (10 µM) into the hypothalamus indicated that a single microinjection can produce a long term increase in CORT concentration in the hypothalamus (Figure 3). This might be due to the local direct injection of CORT into PVN regions. Local injection might delay the drug absorption and led to a sustainable high concentration of drug in the microenvironment. Therefore, 28 days after infusion, the slight elevation of CORT in the hypothalamus still inhibited HPA axis activity and therefore reduced CORT level in the plasma (Figure 4J). DMSO is a type of common solvent with low toxic potential. To exclude the possibility that the toxic potential of DMSO account for the effect of DMSO on depressive-like behaviors and the HPA axis activity, we investigated the effect of DMSO. The experiments demonstrated that the effect of CORT did not attribute to DMSO interference (Figure 2G–K).

In summary, our research imply that the role of the excessive glucocorticoids arriving at hypothalamus after stress is not contributing to depressive-like behaviors and hyperactivity of HPA axis and may be just participating in the feedback inhibition modulation of HPA axis. In addition, although the acute exposure to glucocorticoids in the hippocampus exert feedback inhibition modulation of HPA axis, the chronic excessive glucocorticoids in the hippocampus are sufficient to induce depressive-like behaviors and the hyperactivity of HPA axis, and account for chronic stress-induced depressive-like behaviors and hyperactivity of HPA axis. Although it remains to be elucidated whether hypothalamus involves in other pathological changes of depression, this work would shed light on the recognition of the roles of the hypothalamus and hippocampus in the stress-induced pathology of depression.
